# Polysaccharide-Rich Fractions from *Ganoderma resinaceum* (Ganodermataceae) as Chemopreventive Agents in N-Diethylnitrosamine-Induced Hepatocellular Carcinoma in Wistar Rats

**DOI:** 10.1155/2022/8198859

**Published:** 2022-04-12

**Authors:** Marius Trésor Kemegne Sipping, Francine Kengne Mediesse, Larissa V. Kenmogne, Judith Emery Ngomoyogoli Kanemoto, Dieudonné Njamen, Thaddée Boudjeko

**Affiliations:** ^1^Laboratory of Phytoprotection and Valorization of Genetic Resources, Biotechnology Centre, Nkolbisson, University of Yaoundé 1, P.O. Box 17673, Etetak, Yaoundé, Cameroon; ^2^Department of Animal Biology and Physiology, Faculty of Science, University of Yaoundé 1, P.O. Box 812, Yaoundé, Cameroon; ^3^Centre for Research on Medicinal Plants and Traditional Medicine, Institute of Medical Research and Medicinal Plant Studies, P.O. Box 13033, Yaoundé, Cameroon; ^4^Department of Surgery, Faculty of Health Sciences, University of Witwatersrand, Johannesburg 2193, South Africa; ^5^Genomics Laboratory, Department of Biological Sciences, Birla Institute of Technology & Science, Pilani, Hyderabad Campus, Jawahar Nagar, Shamirpet Mandal, Hyderabad, India; ^6^Department of Biochemistry, Faculty of Science, University of Yaoundé 1, P.O. Box 812, Yaounde, Cameroon

## Abstract

Hepatocellular carcinoma (HCC) is one of the most common and lethal diseases worldwide. Its treatment remains ineffective and the prognosis remains severe, thus favoring the emergence of a preventive approach. Mushroom-derived polysaccharides offer great opportunities because of their less toxicity and bioactivities. The present study aimed to investigate the chemopreventive effects of water-soluble polysaccharides from *Ganoderma resinaceum* on HCC. Two *G. resinaceum* polysaccharide-rich fractions (GRP I and GRP II) were obtained following hot water and alcohol precipitation. Their proteins, phenol compounds, and total neutral sugar content were assayed. The *in vitro* antiproliferative effect was assessed in MDA-MB 231, Hela, and HepG_2_ using the MTT assay. Further, for the *in vivo* study, seven groups of nine rats each received N-diethylnitrosamine (100 mg/kg BW), vehicle (NaCl 0.9%), doxorubicin (10 mg/kg BW), or *G. resinaceum* polysaccharides (125 and 250 mg/kg BW). Liver cancer initiation and progression was assessed by evaluating histomorphology of liver section, hepatic injury markers, hematology, cytokines/chemokines levels, and stress oxidative markers. GRP II presented higher protein and sugar and lower phenol compound content than GRP I. GRP exhibited CC_50_ of 340 and 261.7 in HepG_2_ cells after 48 h. Moreover, GRP I and GRP II (125 and 250 mg/kg) prevented the alteration of the histoarchitecture of the liver induced by the DEN. Alanine aminotransferase (ALT), aspartate aminotransferase (AST), alpha-fetoprotein (AFP), proinflammatory cytokines (G-CSF, IFN*γ*, and TNF*α*), and chemokines (eotaxin and fractalkine) levels were significantly decreased in the GRP I- and GRP II-treated groups, while anti-inflammatory cytokines (IL-10 and IL-12p70) levels were increased. The antioxidant defense was also stimulated by reducing malondialdehyde (MDA) and nitric oxide (NO_2_) levels, increasing catalase (CAT) and superoxide dismutase (SOD) activities, and reducing glutathione (GSH) levels. Our results indicate that GRP I exhibits chemopreventive effects by inhibiting cell proliferation and restoring liver architecture, antioxidant enzymes, and cytokines/chemokines balance.

## 1. Introduction

The prevalence of liver cancers is increasing in Asian and African populations due to their exposure to hepatitis B or C virus, the foremost causative agent of hepatocellular carcinoma (HCC) [1]. HCC is one of the most frequent solid malignancies, accounts for 80–90% of liver cancers, and is the third common cause of cancer morbidity, resulting in almost one million deaths every year [2]. The survival rate after diagnosis of this disease is very low (∼5%), as HCC is often diagnosed at a later stage [3]. Etiological factors of HCC include alcoholism, cirrhosis, hepatic steatosis, and some environmental chemicals. Nitrosamines, for instance, are an important class of environmental carcinogens owing to their carcinogenic and mutagenic properties [4].

Despite the plethora of conventional methods of treatment of HCC, such as chemotherapy, intervening therapy, and surgical resection, there is a lack of diagnostic tools leading to impoverishing their clinical benefits. Moreover, the drugs used in the treatment of HCC are associated with hepatotoxicity, and side effects have been observed with drugs such as 5-fluorouracil, cisplatin, and doxorubicin [5]. In fact, due to the highly vascular nature of the liver, HCC is prone to intrahepatic and extrahepatic metastases leading to treatment failure. Therefore, to manage hepatocellular carcinoma, there is an urgent need to develop less toxic and more efficient therapies targeting oxidative stress, inflammation, and tumor cells' proliferation [6–9]. Polysaccharide-rich fractions from natural plants and mushrooms due to their less toxicity and biological activities have recently attracted the attention of the scientific community. Many reports have highlighted anticancer activities of polysaccharide-rich fractions from mushroom species, namely, *Astragalus membranaceus, Salvia miltiorrhiza, Grifola frondosa, Angelica sinensis,* and Huaier fungus at the doses 60, 120, 180, and 240 mg/kg [10–13]. Moreover, intragastric administration of *Ganoderma lucidum* polysaccharide (GLPS) at 100, 200, and 400 mg/kg for 09 consecutive days significantly decreases the tumor weight in a dose-dependent manner [14]. Many studies have reported hepatoprotective effects in HCC of some species belonging to the Ganodermataceae family due to their bioactive substances, including triterpenoids, sterols, steroids, peptides, and polysaccharides. It has been shown that *G. lucidum* polysaccharides (GLP) could inhibit tumor growth *in vitro* and *in vivo* either by directly blocking the cell cycle of hepatocarcinoma cells (HepG_2_, BEL-7402, and Huh-7) or by indirectly regulating the immune system [15–17]. GLP could suppress HepG_2_ cells via regulating hepatic miRNAs and immune-related miRNAs [18]. Yu et al. [19] have revealed that GLP could be a potent radiation sensitizer in HCC treatment. Otherwise, *G. resinaceum* has also proven its efficacy against liver injury induced by hepatotoxic agents. Peng et al. [20] have found that various terpenes from *G. resinaceum* had significant hepatoprotective activities due to their remarkable *in vitro* inhibitory activities against the increase of ALT and AST levels in HepG_2_ cells induced by H_2_O_2_. The anticancer mechanisms involved include induction of tumor cell apoptosis, immunopotentiation activity in combination with chemotherapy, and inhibition of tumor cell growth and metastasis [21].

Our previous studies have revealed that, at 125 mg/kg, GRP I and GRP II exhibited *in vivo* anti-inflammatory activities by limiting the infiltration of immune cells in the subcutaneous tissues, downregulating the production of proinflammatory cytokines (G-CSF, IFN*γ*, and TNF*α*) and chemokines (eotaxin and fractalkine), and upregulating the production of anti-inflammatory cytokines (IL-10 and IL-12p70) [22].

Nevertheless, so far, no studies have targeted the anticancer activity of the polysaccharide-rich fractions from *Ganoderma resinaceum* fruiting bodies. Therefore, the present work thus aimed to highlight the chemopreventive effects of *G. resinaceum* polysaccharide-rich fractions in the N-diethylnitrosamine-induced hepatocellular carcinoma model.

## 2. Materials and Methods

### 2.1. Chemicals and Reagents

Chemical reagents for antioxidant assays were purchased from GIBCO (Grand Island, NY, USA). The N-diethylnitrosamine (CAS 55-18-5) was purchased from Tokyo Chemical Industry (Tokyo, Japan). Doxorubicin hydrochloride was supplied by Accord Healthcare France SAS (Lille, France). The rat alpha-fetoprotein (AFP) was obtained from Elabscience Biotechnology Inc. (USA). Trypan Blue (0.4%), [3-(4, 5-dimethyl-thiazol-2-yl)-2, 5-diphenyltetrazolium bromide], and cell culture media (RPMI and MEM) were from Sigma Aldrich (St. Louis, MO, USA). All solutions and buffers were prepared in Ultrapure Milli-Q water. MDA-MB 231, Hela, and HepG_2_ were obtained from the National Centre for Cell Science (NCCS) in Pune, India.

### 2.2. Collection and Pretreatment

*Ganoderma resinaceum* fruiting bodies were harvested in the locality of Mbalmayo (Centre Region of Cameroon) in May 2016. A sample was identified in comparison to the Voucher specimen DM 764 at the Laboratory of Mycology of the University of Yaoundé I, Cameroon. The mushrooms were then cut into small pieces, air-dried in the shade at room temperature, and powdered.

### 2.3. Isolation of Soluble Polysaccharides

*G. resinaceum* polysaccharide-rich fractions (GRP) were obtained as described by Hua et al. [23] with slight modifications. Briefly, the powder (650 g) was sieved and incubated in 6 L of 50% methanol at room temperature for 24 h. Then, the mixture was filtered using Whatman paper N° 10 and dried at 40°C for 2 h. The residue in distilled water 1 : 15 (w/v) was boiled at 80°C for 2 h and filtered with Whatman paper N° 10. The polysaccharides were precipitated by stepwise addition of ethanol to concentrations of 60 and 80%. After centrifugation (5000 g, 20 min), the pellet was resuspended in distilled water, dialyzed against distilled water (MWCO 14.000), and freeze-dried for further analysis. The polysaccharide-rich fractions precipitated by 60 and 80% were termed GRP I and GRP II, respectively.

### 2.4. Phytochemical Analysis

 
*Protein Content Assay.* The quantity of proteins present in each polysaccharide-rich fraction was determined by the Bradford method using Bovine Serum Albumin (BSA) as standard [24]. Briefly, each polysaccharide-rich fraction (1 mL) was added to the same volume of Bradford reagent freshly prepared. After incubation in the darkness for 30 min, the absorbance of the mixture was measured at 595 nm using a UV-VIS 1605 Shimadzu spectrophotometer.
 
*Total Phenol Compounds Content Assay.* The phenol compounds were quantified in the polysaccharide-rich fractions using the Folin–Ciocalteu (FC) reagent [25]. The FC reagent (75 *µ*L) was added to 750 *µ*L of polysaccharide-rich fractions (1 mg/mL). After 3 min incubation at room temperature, 750 *μ*L of Na_2_CO_3_ (20%) was added, and the mixture was incubated for 30 min in the darkness. The absorbance was read at 760 nm using a UV-VIS 1605 Shimadzu spectrophotometer. The amount of total phenols was estimated as expressed as ferulic acid equivalent/mg of dry weight (FAE/mg DW).
 
*Determination of Total Sugar Content.* The quantification of total sugars was performed using the phenol-H_2_SO_4_ colorimetric method with glucose as standard [26]. The total sugars were heated in an acid medium to release monosaccharides, which are transformed into dehydrated derivatives of furfural. In each tube containing 0.2 mL of polysaccharide-rich fractions, 0.2 mL of 5% phenol and 1 mL of concentrated sulfuric acid were added. The mixture was stirred and placed at 100°C for 10 min. Then, the mixture was cooled down, and the absorbance was read at 485 nm. The amount of sugar was expressed as *μ*g equivalent glucose (EG)/mg of dry polysaccharide.

### 2.5. Cytotoxicity Assay

 
*Cell Culture.* MDA-MB 231, an ER-adenocarcinoma breast cell, Hela, a cervical cancer cell, and HepG_2_, a liver cancer cell, were obtained from the National Centre for Cell Science (Pune, India). MDA-MB 231 cells were cultured in Leibovitz (L-15) medium, while HepG_2_ cells and Hela cells were cultured in Minimum Essential Minimum Eagle (MEM) and Roswell Park Memorial Institute medium (RPMI), respectively. All cell cultures were supplemented with 10% fetal bovine serum (FBS), L-glutamine (200 mM), streptomycin (200 *μ*g/mL), and penicillin (200 U/mL) and maintained at 37°C in 5% CO_2_. The assay was carried out on cells with 85% confluence.
 
*MTT Assay.* About 10^3^ cells/100 *µ*L/well were plated in 96-well plates and incubated overnight in appropriate cells culture conditions. Then, they were treated with GRP I and GRP II diluted at various concentrations of 125–1000 *µ*g/mL with an appropriate cell culture medium for 48 hours in 5% CO_2_ at 37°C. Thereafter, 20 *µ*L of MTT (5 mg/mL in PBS 1X) was added to each well, followed by an additional incubation of 4 hours. The supernatant was discarded, and formazan blue, which was formed in the cells, was dissolved with 100 *µ*L of dimethyl sulfoxide (DMSO). The plates were further incubated for 3 hours. The optical density was measured at 630 nm using a microplate ELISA reader (INNO-M, LTEK Corporation, South Korea).

The growth inhibition percentage was determined using the following formula:

(1) $$\begin{array}{c} \%\text{ of inhibition of cell proliferation}=1-\left[\left(\frac{\text{OD}\left(630\text{ nm}\right)\text{test}}{\text{OD}\left(630\text{ nm}\right)\text{control}}\right)\right]\times 100, \end{array}$$

where OD control was the absorbance of the control and OD test was the absorbance in the presence of the sample.

### 2.6. *In Vivo* Experiment

 
*Animal's Preparation*. Healthy male Wistar rats aged 5 weeks and weighing 65–80 g were obtained from the breeding of the laboratory of Animal Physiology and Biology (University of Yaoundé 1). The rats were housed in polypropylene cages at room temperature, supplied with standard rat chow and water ad libitum, and kept at a natural light/dark cycle. The experiments were conducted in accordance with the principles and procedures of the European Union Animal Care (CEE Council 86/609) guidelines adopted by the Cameroon Institutional National Ethics Committee, Ministry of Scientific Research and Innovation (Reg. number FWA-IRD 0001954).
 
*Induction of Hepatocarcinoma.* The rats were acclimatized for one week prior to being used. Then, they were randomly divided into 7 groups of 9 rats, each scheduled as the normal group and the negative control group, and received NaCl 0.9% only per os as a vehicle; the positive control group received doxorubicin as a reference drug at a dose of 10 mg/kg once a week *i.p.* and four test groups have received GRP I and GRP II at the doses of 125 and 250 mg/kg BW per day, respectively. The later dose of doxorubicin is equivalent to the human dose of 20 mg/m^2^ according to Barnes and Paget [27]. Two weeks after this dietary regimen, all groups except the normal group were administered with DEN (100 mg/kg BW, i.p.) dissolved in NaCl 0.9% once a week around 4 : 00 pm; phenobarbital (PB) 0.1% was introduced in drinking water as a promoter for 10 weeks. The normal group was also injected with an equal volume of vehicle for successive 10 weeks. The body weight and the behavioral changes of animals were recorded weekly. The promoter was withdrawn from drinking water for the last 3 days, and the animals that were dead or became moribund during the experiment were autopsied and sacrificed. After 12 weeks of experimentation, the remaining animals were sacrificed by cervical decapitation after 12 h of nonhydric fasting and valium (10 mg/kg BW) and ketamine anesthesia (50 mg/kg BW). The blood was collected in anticoagulant (EDTA) tubes for hematological study and in dried tubes (centrifuged at 600 g for 15 min at 4°C) for biochemical assays. Furthermore, liver was excised, washed in ice-cold saline, and blotted to dryness. A part of liver sections was fixed in a 10% neutral formalin solution for histomorphological analysis. Homogenate (1%) of liver tissue was prepared in Tris-HCl (0.1 M; pH 7.4) and centrifuged (600 g, 15 min), and the supernatant was used for biochemical assays. Organs such as brain, lungs, kidneys, spleen, and adrenergic glands were also removed and weighed.
 
*Histomorphological Analysis*. The pieces of liver were dehydrated in increased concentrations of ethanol solution and embedded in paraffin prior to sectioning 5-6 *μ*M thickness with the rotating microtome (Leitz 1512, Marshall Scientific, Hampton, USA). The liver slices were stained with hematoxylin-eosin (HE), and histomorphological modifications were observed under Axioskop 40 microscope. Hepatocellular inflammatory infiltration and cell death were assessed.
 
*Biochemical Analysis*. The liver enzymes, aspartate (AST), and alanine (ALT) transaminases activities were determined using kits from Fortress Diagnostics Limited (Muckamore, United Kingdom). Alpha-fetoprotein (AFP) level was determined using ELISA kit (E-EL-R0153, Elabscience, Houston, USA) with respect to the manufacturer's instructions.
 
*Multiplex Cytokines Assay*. Serum samples from experimental rats were analyzed for cytokines and chemokines using the Rat Cytokine/Chemokine Magnetic Bead Panel (EMD Millipore Corporation, Massachusetts, USA) according to the manufacturer's instructions. Briefly, 25 *µ*L of 1 : 2 diluted samples and standards (1 : 4 serial dilutions) was added in appropriate wells. Then, 25 *µ*L of magnetic beads in solution was added, and the plate was washed twice with 1X wash buffer. The plate was incubated on an orbital shaker at 1000 rpm for 1 hr after adding 25 *µ*L of detection antibodies; streptavidin-phycoerythrin (25 *µ*L) was added, followed by an incubation of 30 min at RT. After washing, plate was resuspended in 125 *µ*L of sheath fluid and read on the MAGPIX® instrument (Luminex, USA). Data obtained was analyzed using the Luminex xPONENT® multiplex assay software.

### 2.7. Stress Oxidative Markers' Assay

 
*Malondialdehyde (MDA) Assay.* MDA is one of the end products in the lipid peroxidation process. The lipid peroxides were estimated in the liver homogenates using the thiobarbituric acid (TBA) reactive substances tests [28]. Tissue homogenates (0.1 mL) and TBA (0.4 mL) were heated at 100°C for 15 min. After cooling rapidly, the mixture was centrifuged at 3000 g for 5 min, and the optical density of supernatant was read at 532 nm against an appropriate blank (without sample). The MDA content was determined using extinction molar coefficient (0.153 *µ*M^−1^ cm^−1^) and expressed as nmol MDA/g protein.
 
*Catalase (CAT) Activity.* Catalase activity was assessed by measuring the degradation of peroxide hydrogen (H_2_O_2_) following the method described by Sinha [29] with slight modifications. The mixture includes 0.2 mL of phosphate buffer (0.0 1 M, pH 7), 0.2 mL of tissue homogenate, and 0.2 mL of hydrogen peroxide. The reaction was stopped after 1 min by adding 1 mL of acetate dichromate, and the mixture was reacted at 100°C for 10 min. After cooling, absorbance was read at 620 nm, and the catalase activity was expressed as U/mg protein, with one unit of catalase activity equal to 1 *μ*mol H_2_O_2_ degraded per minute.
 
*Superoxide Dismutase (SOD) Assay.* Superoxide dismutase activity was determined as described by McCord and Fridovish [30] with slight modifications based on the inhibition of adrenalin's autoxidation. The reaction mixture consisted of 0.2 mL of sample and 2.5 mL of carbonate buffer (0.05 M, pH 10.2). The reaction was initiated by adding 0.3 mL of adrenalin freshly prepared in buffer solution. The developed blue color was read at 480 nm at room temperature for 3 min. Units of SOD activity were expressed as the amount of enzyme required to inhibit autoxidation of adrenalin by 50%, and it was expressed as U/mg protein.
 
*Glutathione Reduced (GSH) Assay*. The levels of GSH in liver homogenates were estimated using the method illustrated by Ellman [31]. The tissue homogenate (in 0.05 M Tris-HCl, pH 7.4) is taken and added with 750 *µ*L of Ellman's reagent (5 mg of dinitro-2,2′-dithio-5,5-dibenzoic in 250 mL of 0.1 M phosphate buffer, pH 6.5). The test tubes were then shaken vigorously and incubated at room temperature for 1 h. Further, optical density was determined against a blank (without sample). The amounts of GSH content were determined using extinction molar coefficient (13600 M^−1^ cm^−1^) and expressed as *µ*mol GSH/g protein.
 
*Nitric Oxide (NO) Content Assay.* The amounts of NO in liver homogenates were determined using the method described by Marcocci et al. [32]. In the test tubes, 500 *µ*L of Griess reagent was added. The mixture was incubated in the darkness at room temperature for 10 min, and absorbance was read at 546 nm against a blank. Sodium nitrite at various concentrations (0.031, 0.062, 0.125, 0.25, 0.5, and 1 *µ*M) was used as standard. The concentrations of NO were determined using a standard curve and expressed as *µ*mol/g.

### 2.8. Hematological Analysis

Hematological parameters (white blood cell (WBC) count, lymphocytes, monocytes, granulocytes, red blood cell (RBC) count, hematocrit, hemoglobin, mean corpuscular hemoglobin (MCH), mean corpuscular hemoglobin concentration (MCHC), mean corpuscular volume (MCV), and platelets) were also evaluated using Mindray BC 2800 Auto Hematology Analyzer form Shenzhen Mindray Bop-Medical Electronics Co., Ltd.

### 2.9. Statistical Analysis

Data were expressed as mean ± standard deviation (SD) for each experimental group. Statistical analysis was performed using GraphPad Prism software version 5.03 (San Diego, CA, USA) using the one-way analysis of variance (ANOVA) followed by Dunnett's post hoc test for multiple comparisons. *P* < 0.05 was considered to be significant.

## 3. Results

### 3.1. Phytochemical Content

**Figure 1:**
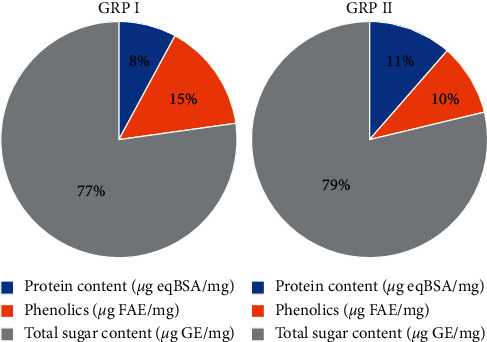
Phytochemical content of GRP I and GRP II.

In this study, two polysaccharide-rich fractions named GRP I and GRP II were obtained from *Ganoderma resinaceum* fruiting bodies using hot water extraction and alcohol precipitation. The partial characterization showed that GRP I and GRP II are mostly linked to proteins and total phenol compounds (Figure 1). Notably, GRP II presented higher protein content (11%) than GRP I (8%). However, the total phenol compounds were lower in GRP II (10%) than GRP I (15%), while total sugars were more abundant in GRP II (79%) than GRP I (77%).

### 3.2. Cytotoxicity Assessment

The cytotoxicity of *G. resinaceum* polysaccharide-rich fractions (GRP) on MDA-MB 231, Hela, and HepG_2_ cancer cells is depicted in Table 1. GRP I and GRP II exhibited cytotoxicity after 48 h of incubation. However, GRP II presented the most significant inhibitory cytotoxic activity with CC_50_ of 298, 341.1, and 298.7 on MDA-MB 231, Hela, and HepG_2,_ respectively.

### 3.3. Effects of GRP on Body Weight and Relative Organ Weights

**Figure 2:**
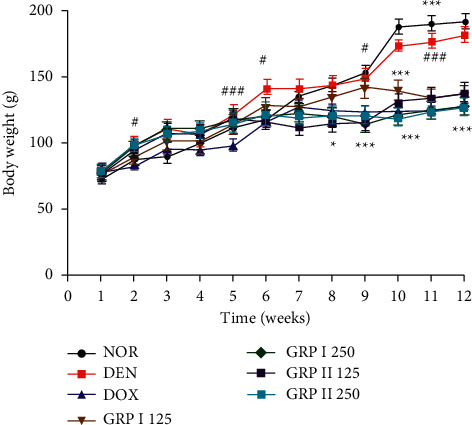
Effects of GRP on body weight of different experimental groups of rats after 12 weeks. NOR = normal control (animals received only saline solution 0.9%); DEN = negative control (animals received N-diethylnitrosamine + saline 0.9%); DOX + DEN = positive control (animals received doxorubicin + N-diethylnitrosamine); GRP I + DEN = animals received polysaccharide fraction GRP I + N-diethylnitrosamine at doses 125 and 250 mg/kg; GRP II + DEN = animals received polysaccharide fraction GRP II + N-diethylnitrosamine at doses 125 and 250 mg/kg. Values represent means ± SD (*n* = 9). All experimental animals except the normal group (NOR) were exposed to a daily dose of N-diethylnitrosamine (100 mg/kg) for 10 weeks. ^###^*P* *<* *0.001* and ^##^*P* *<* *0.01* compared to the normal group; ^*#*^*P* *<* *0.05* compared to the normal group; ^*∗*^*P* < 0.05 compared to the negative control; ^*∗∗*^*P* < 0.01 compared to the negative control; ^*∗∗∗*^*P* < 0.001 compared to the negative control.

All the treatments significantly affected the animal body weights throughout the experiment. However, doxorubicin-treated rats have presented lower weights (*P* < 0.01) as compared to the normal group (Figure 2). The treatments with GRP I and GRP II significantly decreased the relative weights of the organs after 12 weeks of treatment. DEN induction led to a significant increase of the relative weights of all organs removed, with the exception of adrenal glands and heart, in comparison to the normal group (*P* < 0.01). Animals treated with polysaccharide fractions GRP I 125 have also significantly reduced the relative weights of liver, kidneys, and spleen (*P* < 0.001) compared to the DEN group; meanwhile, GRP II 125 has presented the highest percentage of reduction. The same observations were done in doxorubicin-treated animals. No significant change was found between DEN and the other treated groups (Table 2).

### 3.4. Histopathological Data

**Figure 3:**
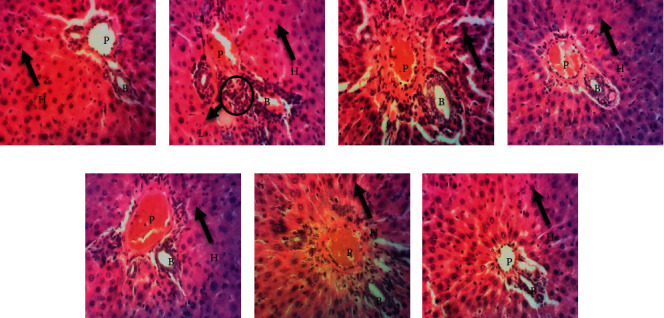
Photomicrographs of liver tissue sections of the experimental groups (X200, HE). (a) The normal group shows an intact cell membrane and normal hepatocytes. (b) Massive infiltration of inflammatory cells (Kupffer cells; leukocyte). (c) Positive control shows a significant restoration of the normal architecture of the liver tissue section with a weak infiltration of inflammatory cells. (d) Animals received polysaccharide fraction GRP I + N-diethylnitrosamine at 125 mg/kg. (e) Animals received polysaccharide fraction GRP I + N-diethylnitrosamine at 250 mg/kg. (f) Animals received polysaccharide fraction GRP II + N-diethylnitrosamine at 125 mg/kg. (g) Animals received polysaccharide fraction GRP II + N-diethylnitrosamine at 250 mg/kg. In the GRP-treated groups (D-G), there was a significant restoration of the normal architecture of liver tissue with radially arranged hepatocytes similar to normal control. He = hepatocyte, Bc = biliary canaliculus, Pv = portal vein, and Li = leukocyte inflammation.

Liver tissue presented hepatic cells with granulated cytoplasm, portal vein, hepatic artery, small uniform nuclei, and nucleolus. So, no normal architecture of liver tissues was observed in the normal group (Figure 3(a)). DEN-treated rats presented a massive infiltration of inflammatory cells: Kupffer cells and leukocytes (Figure 3(b)). Architecture of liver tissue sections of doxorubicin-treated rats was improved in comparison to the negative control rats. Nevertheless, a weak infiltration of inflammatory cells was observed (Figure 3(c)). Significant restoration of architecture was observed in liver tissue of the GRP-treated groups as compared to the negative control group characterized by an absence of inflammatory cells in portal veins and normal appearance of liver cells (Figures 3(d)–3(g)).

**Figure 4:**
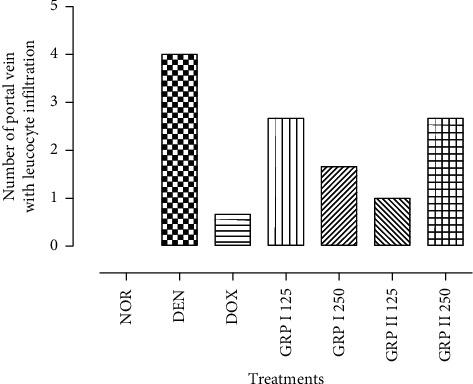
Number of portal veins with infiltration of immune cells in the experimental groups.

Histopathological findings were confirmed by the number of portal veins presenting infiltration of immune cells. The normal group has presented any leukocyte infiltration. Meanwhile, the negative control group has shown 04 portal veins with immune cells infiltration. On the other hand, positive control has presented the lowest numbers of portal veins with infiltration of immune cells (0.67). GRP treated groups at 125 and 250 mg/kg have shown slightly infiltration of immune cells in portal veins. (Figure 4).

### 3.5. Effects of GRP on Some Biochemical Markers of Hepatocellular Carcinoma

**Figure 5:**
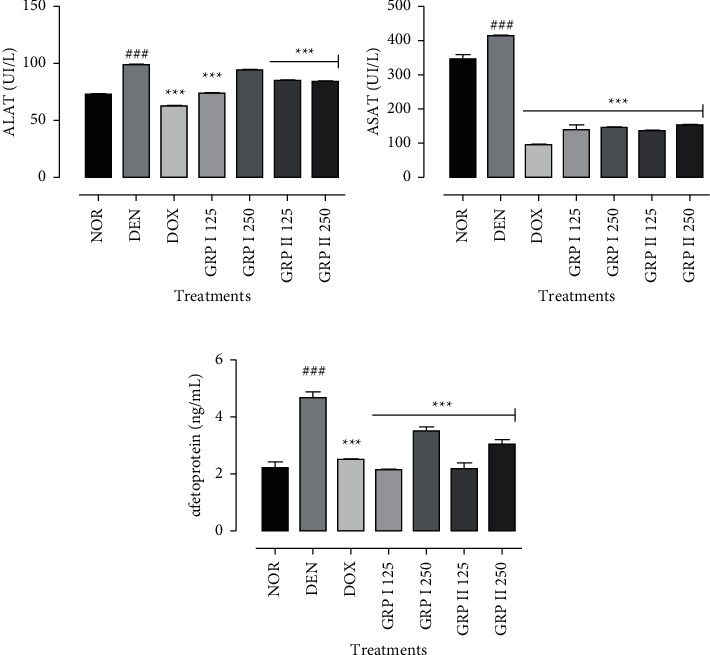
Effects of GRP on ALAT (a), ASAT (b), and AFP (c) levels. NOR = normal control (animals received only saline solution 0.9%); DEN = negative control (animals received N-diethylnitrosamine + saline 0.9%); DOX + DEN = positive control (animals received doxorubicin + N-diethylnitrosamine); GRP I + DEN = animals received polysaccharide fraction GRP I + N-diethylnitrosamine at doses 125 and 250 mg/kg; GRP II + DEN = animals received polysaccharide fraction GRP II + N-diethylnitrosamine at doses 125 and 250 mg/kg. Values represent means ± SD (*n* = 9). All experimental animals except the normal group (NOR) were exposed to a daily dose of N-diethylnitrosamine (100 mg/kg) for 10 weeks. ^###^*P* < 0.001 and ^##^*P* 0.01 compared to the normal group; #*P* < 0.05 compared to the normal group; ^*∗*^*P* < 0.05 compared to the negative control; ^*∗∗*^*P* < 0.01 compared to the negative control; ^*∗∗∗*^*P* < 0.001 compared to the negative control.

Exposition to DEN led to significantly (*P* < 0.001) increased ALT and AST activities by 36 and 19%, respectively, as compared to the normal groups. Significant (*P* < 0.001) decreases have been noticed in the doxorubicin group and the GRP I- and GRP II-treated groups as compared to the negative control rat (Figures 5(a) and 5(b)). According to AFP, there was a significant increase in HCC rats treated with DEN in comparison to the normal group (*P* < 0.001). Doxorubicin administration significantly reduced AFP levels by 1.9-fold as compared to the negative control (*P* < 0.001). GRP I and GRP II have also reduced AFP levels at 125 and 250 mg/kg (by 2.2, 1.3, 2.1, and 1.5-fold), respectively, compared to the negative control (Figure 5(c)).

### 3.6. Effects of GRP on Cytokines and Chemokines Production

**Figure 6:**
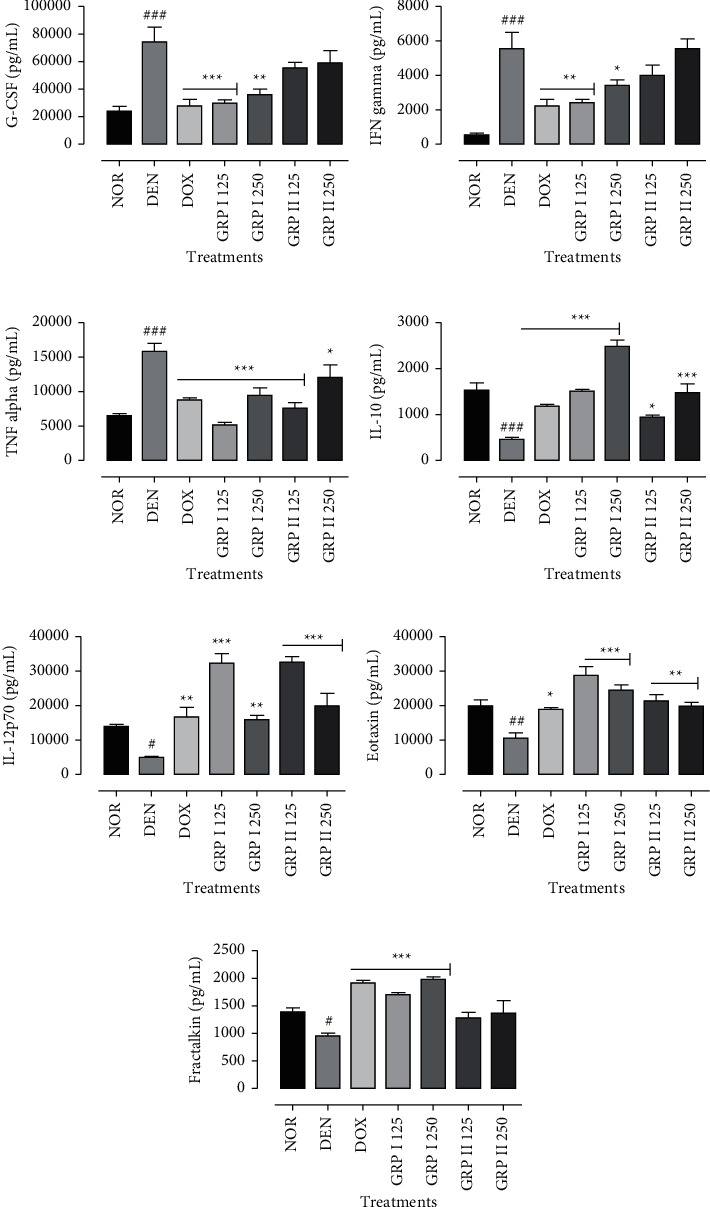
Effects of GRP on serum cytokines and chemokines levels. NOR = normal control (animals received only saline solution 0.9%); DEN = negative control (animals received N-diethylnitrosamine + saline 0.9%); DOX + DEN = positive control (animals received doxorubicin + N-diethylnitrosamine); GRP I + DEN = animals received polysaccharide fraction GRP I + N-diethylnitrosamine at doses 125 and 250 mg/kg; GRP II + DEN = animals received polysaccharide fraction GRP II + N-diethylnitrosamine at doses 125 and 250 mg/kg. The following cytokines, namely, (a) G-CSF, (b) IFN*γ*, (c) TNF*α*, (d) IL-10, (e) IL-12 p70, (f) eotaxin, and (g) fractalkine, were analyzed. One-way ANOVA and Bonferroni test were used to compute statistics between groups, respectively. Values represent means ± SD (*n* = 9). All experimental animals except the normal group (NOR) were exposed to a daily dose of N-diethylnitrosamine (100 mg/kg) for 10 weeks. ^###^*P* < 0.001 and ^##^*P* < 0.01 compared to the normal group; ^#^*P* < 0.05 compared to the normal group; ^*∗*^*P* < 0.05 compared to the negative control; ^*∗∗*^*P* < 0.01 compared to the negative control; ^*∗∗∗*^*P* < 0.001 compared to the negative control.

In general, DEN has caused an overproduction of proinflammatory cytokines: G-CSF, IFN*γ*, TNF*α* (*P* < 0.001), eotaxin (*P* < 0.01), and fractalkine (*P* < 0.05) compared to the normal groups. However, GRP leads to a significant decrease in proinflammatory cytokines and chemokines as positive control (DOX). The more significant effects were observed in the GRP I 125-treated group with 30000 ± 5773.50, 2500 ± 182.57, and 5247.79 ± 549.85 for G-CSF, IFN*γ*, and TNF*α*, respectively (Figures 6(a)–6(c)). GRP I 125 also has the highest effects on eotaxin and fractalkine levels with 28449.86 ± 4523.66 and 1686.22 ± 62.64 pg/mL (Figures 6(f)and 6(g)). On the other hand, the levels of antitumor cytokines (IL-10 and IL-12p70) were significantly (*P* < 0.001) decreased in the GRP-treated group compared to the negative control (DEN). The highest values were obtained in GRP I 250 for IL-10 and in GRP I 125 with 2524.49 ± 312.67 and 31480.93 ± 57.84 pg/mL, respectively (Figures 6(d) and 6(e)).

### 3.7. Oxidative Stress Markers

**Figure 7:**
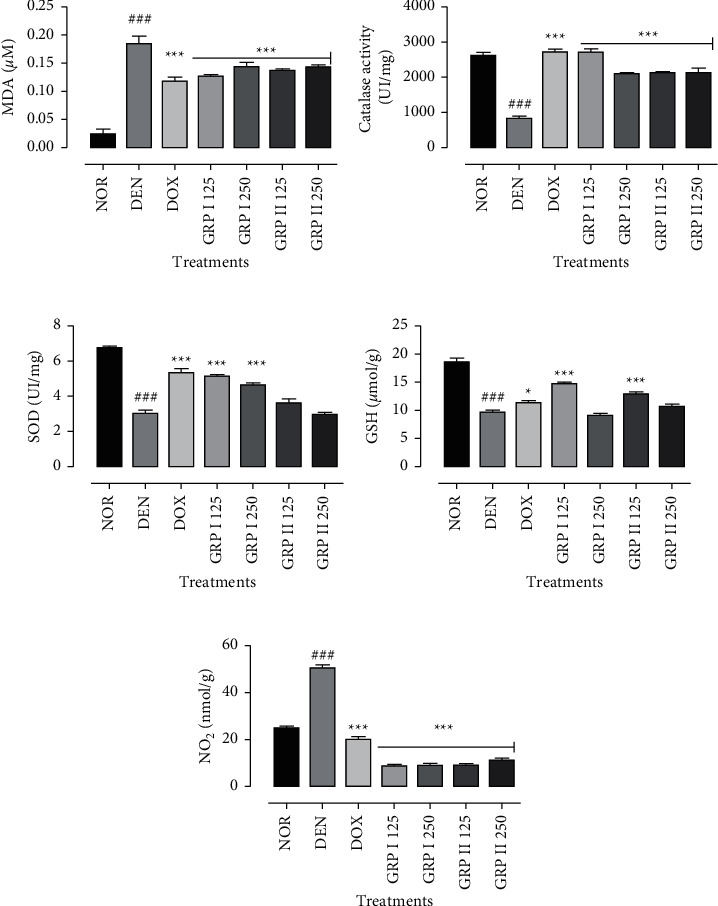
Effects of *G. resinaceum* polysaccharides on enzymatic and nonenzymatic parameters. (a) MDA levels. (b) Catalase activity. (c) SOD activity. (d) GSH levels. (e) NO_2_ production. NOR = normal control (animals received only saline solution 0.9%); DEN = negative control (animals received N-diethylnitrosamine + saline 0.9%); DOX + DEN = positive control (animals received doxorubicin + N-diethylnitrosamine); GRP I + DEN = animals received polysaccharide fraction GRP I + N-diethylnitrosamine at doses 125 and 250 mg/kg; GRP II + DEN = animals received polysaccharide fraction GRP II + N-diethylnitrosamine at doses 125 and 250 mg/kg. Values represent means ± SD (*n* = 9). All experimental animals except the normal group (NOR) were exposed to a daily dose of N-diethylnitrosamine (100 mg/kg) for 10 weeks. ^###^*P* < 0.001 and ^##^*P* < 0.01 compared to the normal group; ^#^*P* < 0.05 compared to the normal group; ^*∗*^*P* < 0.05 compared to the negative control; ^*∗∗*^*P* < 0.01 compared to the negative control; ^*∗∗∗*^*P* < 0.001 compared to the negative control.

The effects of *G. resinaceum* polysaccharide-rich fractions on oxidative stress markers are illustrated in Figure 7. DEN induced a significant (*P* < 0.001) increase in malondialdehyde (MDA) and nitric oxide (NO_2_) levels by 6.6- and 2.0-fold, respectively, as compared to the normal control (Figures 7(a) and 7(e)). Moreover, DEN depleted significantly (*P* < 0.001) catalase and superoxide dismutase (SOD) activities and reduced glutathione (GSH) levels by 54.7, 54.5, and 47.8%, respectively, compared to the normal control, while doxorubicin and GRP improved the antioxidant parameters of experimental rats (Figures 7(b)–7(d)). Doxorubicin, GRP I, and GRP II at all doses significantly (*P* < 0.001) reduced the MDA levels when compared to the negative control (Figure 7(a)). Catalase activity significantly increased (*P* < 0.001) by 3.2-fold (DOX and GRP I 125) and 2.5-fold for the GRP I 250-, GRP II 125-, and GRP II 250-treated groups, respectively, with respect to the negative control groups (Figure 7(b)). SOD activity was increased significantly (*P* < 0.001) in the doxorubicin-, GRP I 125-, and GRP II 250-treated groups by 1.8-, 1.7-, and 1.5-fold, respectively (Figure 7(c)). GRP I 125 and GRP II 125 increased significantly (*P* < 0.05) GSH levels by 1.5- and 1.3-fold (*P* < 0.001) more than the DOX-treated group by 1.2-fold (Figure 7(d)). NO_2_ levels were significantly lowered (*P* < 0.001) by the doxorubicin- and GRP-treated groups (Figure 7(e)).

### 3.8. Hematological Analysis

As shown in Table 3, the rats treated with DEN significantly decreased WBCs, monocytes (*P* < 0.001), lymphocytes, granulocytes (*P* < 0.01) counts, and mean corpuscular hemoglobin concentration (MCHC) (*P* < 0.05) in comparison to the normal control group. Animals treated with GRP I at 125 and 250 mg/kg have shown a significant increase in WBCs, lymphocytes (*P* < 0.05) count, and hematocrit (*P* < 0.001) as compared to the negative control group. Furthermore, a significant decrease in monocytes (*P* < 0.001), MCV (*P* < 0.001), granulocytes (*P* < 0.05), and (*P* < 0.001) was observed for 125 and 250 mg/kg, respectively. There was a significant increase in WBCs (*P* < 0.01) and granulocytes counts in GRP II 125 (*P* < 0.01) and GRP II 250 (*P* < 0.05) as compared to the negative control group. Monocytes significantly decreased in the GRP II 125 (*P* < 0.01) and GRP II 250 groups (*P* < 0.05) with respect to the negative control group.

## 4. Discussion

The liver, due to its many functions including the metabolism of carbohydrates, fats, and proteins; biosynthesis of urea and cholesterol; storage of vitamins and minerals; and regulation of glycaemia and removal of xenobiotics from blood (such as alcohol, drugs), is the privileged target of chemical toxicants [33, 34]. Diethylnitrosamine-phenobarbital (DEN/PB) is a suitable experimental model in liver carcinogenesis and chemoprevention, thanks to its well-known metabolic pathway and carcinogenicity [35]. PB is generally used as a promoter of HCC due to its hepatotoxicity through metabolic activation and enhancement of hepatic enzymes, including cytochrome P450s, glucuronosyltransferases, and glutathione S-transferases. In fact, activation of nitrosamines by cytochrome P450 enzymes leads to adducts with DNA and cell proteins and has a high activity on centrilobular hepatocytes [36, 37].

Partial characterization of GRP I and GRP II revealed the presence of phenolics and proteins suggesting glycoproteins structure in mycetes matrix [38, 39].

Cytotoxicity is an appropriate prerequisite in the study of biological properties of pure biocompounds or mixtures. In fact, cytotoxicity appears as a useful tool to indicate the antiproliferative or lethal effects of a substance [40]. *G. resinaceum* polysaccharide-rich fractions exhibited weak cytotoxic effects against MDA-MB 231, Hela, and HepG_2_ with CC_50_ range of 298–420 *µ*g/mL after 48 h of incubation. Polysaccharides are generally known for their *in vitro* anticancer activities either by regulating cell cycle, intracellular Ca^2+^ concentration triggering apoptosis, immunopotentiation, or miR-125b inhibiting T_regs_ accumulation and function [41–46]. These properties might be associated with the specific conformation of polysaccharides and their affinity for cancer cell surface [47]. However, considering the aforementioned arguments and the traditional uses of *G. resinaceum* to manage various ailments, we sought to determine the *in vivo* antitumor activities of *G. resinaceum* polysaccharide-rich fractions.

Histopathological findings in liver specimens are in line with biochemical results. Microscopic analysis of the DEN-treated groups has shown a massive infiltration of inflammatory cells in the portal vein reflecting the initiation of hepatocellular carcinoma. These observations overcame such evidence on DEN/PB carcinogenicity [48, 49]. Liver specimens from GRP-treated rats after DEN administration showed quasinormal microarchitecture with no necrosis, few scattered foci of inflammation in hepatocyte lobule, and nondilated portal veins. Similar observations have been reported on the protective effect of polysaccharides and other natural plants and mushrooms such as *Lycium chinensis*, *Astragalus membranaceus, Antrodia cinnamomea, Lycium barbarum,* and *Mangifera indica* on chemically liver injury [50–54].

Hepatic biomarkers such as transaminases (ALT and AST) and alpha-fetoprotein levels offer insights into liver functions [55]. The abnormal increases of ALT and AST levels in sera imply resultant liver injury induced by DEN. This compound breaks down cell membrane architecture (leading to spillage of these enzymes into serum) which in turn could induce overproduction of these enzymes [56–59]. AFP is a glycoprotein synthesized during early fetal life; its level falls quickly after birth. Elevation of serum alpha-fetoprotein levels is usually characteristic of advanced HCC in adults [60]. This can be explained by its strong implication as a transport molecule for many ligand heavy metals, bilirubin, and many xenobiotics. Moreover, AFP intervenes in the regulation of cell proliferation and immunosuppression [61, 62]. In the current investigation, we have found that later, doxorubicin can also enhance AFP levels. In fact, doxorubicin can cause an idiosyncratic reaction and potentially contribute to liver toxicity by delaying excretion, increasing accumulation of the drug in plasma and tissues, leading to systemic side effects like cardiomyopathy [63]. In contrast, *G. resinaceum* polysaccharides decrease ALT, AST, and AFP levels leading to maintaining the integrity of plasma membrane and suppressing the leakage of enzymes through membranes. These results reflect the ability of GRP to lessen hepatocellular carcinoma initiation and promotion.

Cytokines are low molecular weight polypeptide mediators of cellular communication that are produced and released by different cell types in the liver. They play a key role in the initiation, maintenance, and progression of tumors. The development of tumors is promoted by cytokines G-CSF, IFN*γ*, TNF*α*, IL-10, and IL-12p70 released by neoplastic cells and tumor-associated macrophages (TAM) [61]. Moreover, chemokines such as eotaxin and fractalkine are a superfamily of proinflammatory molecules providing signals for tracking, adhesion, and migration of leukocytes at sites of injury and inflammation [64]. In this study, administration of DEN induced overproduction of proinflammatory cytokines (G-CSF, IFN*γ*, and TNF*α*) and chemokines such as eotaxin and fractalkine, while GRP I alleviated significantly these inflammatory cytokine levels. The levels of IL-10 and IL-12p70 were significantly increased in the GRP-treated groups. Polysaccharides are reported as potent antitumor agents due to their immunoregulatory activities [65, 66]. Chemokines such as fractalkine are key elements in the progression of HCC. The expression of fractalkine and its receptor CX3CR1 is upregulated in hepatocytes during liver injury. The antitumor activities of fractalkine have been reported in several *in vivo* studies using normal mice [67]. Eotaxins are potent chemoattractant cytokines generally involved in allergic diseases. They have also revealed their implications in regulation of other immune cells in tumor microenvironment or direct cytotoxic functions against cancers cells. Studies have shown that TATE (tumor-associated tissue eosinophilia) or degranulation of eosinophilia is connected with an improved prognosis of some types of tumors such as colorectal cancer, esophageal, bladder, or prostate cancer [68].

Furthermore, antioxidant properties of *G. resinaceum* polysaccharides were examined to better understand their hepatoprotective effects. Hepatocellular carcinoma can be triggered by DEN through the overproduction of free radicals reacting with proteins, lipids, and nucleic acids. Biotransformation of DEN by cytochrome P450 produces promutagenic adducts O^6^-ethyl-deoxyguanosine and O^4^- and O^6^-ethyl deoxythymidine that bind to DNA, destabilizing gene sequences. Consequently, these electrophilic compounds overwhelm antioxidant defenses proceeding to oxidative stress and initiating liver carcinogenesis [69–71]. As antioxidant enzymes, SOD converts superoxide radicals to hydrogen peroxide. Hereafter, catalase catalyzes the dismutation of hydrogen peroxide to water and oxygen [72].

DEN treatment led to significant decreases in antioxidant enzymes (SOD and CAT) and GSH and increases in lipid peroxidation. The results are consistent with those reported by Hebatallah et al. [73], whereas these effects are attenuated in GRP-treated rats confirming their hepatoprotective properties by either free radicals scavenging or antioxidant activities. High production of NO and inducible nitric oxide synthase (iNOS) expression is noticed in DEN-induced HCC. The main roles of NO include vasodilatation, inhibition of platelet, cell-to-cell communication, and cytotoxicity beneficial for improving necrosis in liver cells. Overproduction of NO by iNOS-mediated hepatotoxicity is more often indirect by activation of inflammatory cells [74]. The elevated level of NO was reduced by GRP treatment, suggesting that their hepatoprotective effects may be related to the inhibition of NO release. These findings are in agreement with other reports on polysaccharides inhibiting DEN-induced oxidative stress [75].

Hematological parameters and markers play a key role in the systemic inflammatory response and have been correlated to several malignancies. Then, they are very sensitive to detecting the deleterious effects of substances on our health. So, it is primordial to study the variations of hematological parameters in liver cancer patients at regular intervals during treatment [76, 77]. Our data revealed that DEN induced pancytopenia characterized by a statistical decrease in white blood cells (WBCs), lymphocytes, monocytes, granulocytes counts, and mean corpuscular volume (MCV) compared to the normal group. The low concentrations of RBCs and hemoglobin are most common in primary HCC patients: this is called normochromic normocytic anemia [78]. Moreover, leucopenia and thrombocytopenia observed in this study are present in patients with splenomegaly and with a history of bleeding tendencies. The abnormalities of hematological parameters in chronic liver diseases are cross-linked to the damage of bone marrow, and plasma membranes contain high levels of prooxidant products [79, 80]. In opposite, *G. resinaceum* polysaccharide-rich fractions increased WBCs, lymphocytes count, and hematocrit, meaning that they could mitigate or attenuate the harmful effects of anemia and leukocytosis against DEN intoxication.

## 5. Conclusions

Taking together, the present findings support that *G. resinaceum* polysaccharide-rich fractions exhibited chemopreventive effects correlated probably to their phytochemical content and molecular weights. GRP exerted cytotoxic activities against MDA-MB 231, Hela, and HepG_2_. GRP also endowed the capacity to initiate the immune response by regulating inflammatory cytokines and chemokines. Biochemical, histopathological, and hematological data in rats indicated that the alleviated effects of GRP are due to its antioxidant and anti-inflammatory properties. The most relevant activities were obtained in the GRP I- and GRP II-treated groups at 125 mg/kg. This study provides insights for using GRP I as a potential chemopreventive agent in hepatocellular carcinoma.

## Figures and Tables

**Table 1:** Cytotoxicity of *Ganoderma resinaceum* polysaccharide-rich fractions (GRP) in cancer cell lines.

CC_50_ (*µ*g/mL)		
	GRP I	GRP II
MDA-MB 231	420.3	298.0
Hela	407.8	341.1
HepG_2_	340.0	261.7

CC_50_: concentration of *G. resinaceum* polysaccharide fractions leading to 50% of cell viability. Hela, MDA-MB 231, and HepG_2_ were incubated for 48 h with increasing concentrations (125–1000 *µ*g/mL) of GRP, and the cell viability was evaluated by MTT assay. The results are expressed in percentage as the mean ± SD.

**Table 2:** Relative weight of wet organs (g/100 g PC) in different experimental groups after 12 weeks.

Organs (mg/kg)	NOR	DEN	DOX + DEN	GRP I + DEN (mg/kg)		GRP II + DEN (mg/kg)	
				125	250	125	250
Liver	327.4 ± 40.3	433.9 ± 17.5^###^	325.5 ± 35.3^*∗∗∗*^	335.6 ± 32.2^*∗∗∗*^	414.7 ± 42.3	394.9 ± 14.9	446.0 ± 16.7
Kidneys	59.7 ± 3.2	86.8 ± 5.8^###^	73.3 ± 3.1^*∗∗∗*^	75.4 ± 3.4^*∗∗∗*^	75.8 ± 3.4^*∗∗*^	73.7 ± 4.8^*∗∗*^	82.5 ± 4.7
Adrenal glands	2.4 ± 0.5	2.5 ± 0.2	3.7 ± 0.6	3.2 ± 0.4	3.6 ± 0.8	3.4 ± 1.2	4.82 ± 0.2^*∗∗∗*^
Heart	37.2 ± 3.9	38.7 ± 3.7	40.7 ± 4.7	39.1 ± 4.3	40.1 ± 1.4	37.0 ± 4.5	41.6 ± 2.5
Lungs	68.6 ± 0.6	142.8 ± 4.5^###^	136.3 ± 1.7^*∗∗*^	91.3 ± 4.7^*∗∗∗*^	103.4 ± 0.3^*∗∗∗*^	97.7 ± 2.7^*∗∗∗*^	123.7 ± 3.2^*∗∗∗*^
Spleen	36.6 ± 2.3	55.2 ± 1.5^###^	41.6 ± 2.3^*∗∗∗*^	48.8 ± 1.1	62.5 ± 4.8^*∗∗*^	74.4 ± 4.0^*∗∗∗*^	70.4 ± 4.1^*∗∗∗*^
Brain	96.0 ± 2.7	126.5 ± 3.8^###^	125.4 ± 3.0	104.8 ± 7.0^*∗∗∗*^	98.7 ± 5.0^*∗∗∗*^	109.5 ± 0.8^*∗∗∗*^	138.0 ± 6.9^*∗∗*^
Testicles	131.3 ± 8.7	94.1 ± 0.6^###^	123.0 ± 5.4^*∗∗∗*^	132.0 ± 1.6^*∗∗∗*^	158.4 ± 1.4^*∗∗∗*^	101.4 ± 2.6^*∗*^	81.8 ± 2.7^*∗∗∗*^
Seminal vesicles	55.8 ± 4.6	31.0 ± 1.3^###^	7.4 ± 0.7^*∗∗∗*^	10.3 ± 1.0^*∗∗∗*^	38.8 ± 0.7^*∗∗∗*^	17.2 ± 1.2^*∗∗∗*^	5.1 ± 0.3^*∗∗∗*^
Epididymis	49.5 ± 3.3	32.0 ± 2.3^###^	32.5 ± 3.1	36.7 ± 4.3	21.6 ± 0.4^*∗∗∗*^	30.2 ± 1.9	20.1 ± 3.1^*∗∗∗*^
Femur	37.6 ± 2.1	29.9 ± 1.5^###^	39.2 ± 3.5^*∗∗∗*^	43.2 ± 0.6^*∗∗∗*^	30.2 ± 0.7	31.0 ± 1.6	52.2 ± 2.7^*∗∗∗*^

NOR = normal control (animals received only saline solution 0.9%); DEN = negative control (animals received N-diethylnitrosamine + saline 0.9%); DOX + DEN = positive control (animals received doxorubicin + N-diethylnitrosamine); GRP I + DEN = animals received polysaccharide fraction GRP I + N-diethylnitrosamine at doses 125 and 250 mg/kg; GRP II + DEN = animals received polysaccharide fraction GRP II + N-diethylnitrosamine at doses 125 and 250 mg/kg. Values represent means ± SD (*n* = 9). All experimental animals except the normal group (NOR) were exposed to a daily dose of N-diethylnitrosamine (100 mg/kg) for 10 weeks. ^###^*P* < 0.001 and ^##^*P* < 0.01 compared to the normal group; ^#^*P* < 0.05 compared to the normal group; ^*∗*^*P* < 0.05 compared to the negative control; ^*∗∗*^*P* < 0.01 compared to the negative control; ^*∗∗∗*^*P* < 0.001 compared to the negative control.

**Table 3:** Effects of *G. resinaceum* polysaccharide fractions on hematological parameters after 12 weeks of treatment.

Parameters	NOR	DEN	DOX	GRP I + DEN (mg/kg)		GRP II + DEN (mg/kg)	
				125	250	125	250
WBC (×10^3^ *µ*L^−1^)	11.30 ± 0.55	5.45 ± 0.21#	16.23 ± 0.09^*∗∗∗*^	11.60 ± 0.51^*∗*^	12.80 ± 0.10^*∗*^	14.60 ± 0.16^*∗∗*^	15.80 ± 0.85^*∗∗*^
Lymphocytes (%)	77.06 ± 3.43	62.6 ± 3.82^##^	70.45 ± 3.71^*∗∗*^	59.70 ± 2.49	73.30 ± 0.96^*∗*^	65.53 ± 3.60	58.45 ± 2.05
Monocytes (%)	10.12 ± 1.89	19.05 ± 1.62^###^	11.33 ± 0.60^*∗∗∗*^	11.20 ± 0.74^*∗∗∗*^	11.40 ± 0.49 ^*∗∗∗*^	11.70 ± 0.65^*∗∗∗*^	11.65 ± 1.91^*∗*^
Granulocytes (%)	14.17 ± 0.48	21.75 ± 1.48^###^	18.25 ± 1.43^*∗∗*^	23.77 ± 3.18^*∗*^	15.40 ± 0.53^*∗∗∗*^	22.10 ± 0.86^*∗∗*^	29.90 ± 0.14^*∗∗∗*^
RBC (×10^3^ *µ*L^−1^)	5.98 ± 0.35	4.95 ± 0.15	4.66 ± 0.42	6.29 ± 0.28	6.76 ± 0.18	5.33 ± 0.27	5.61 ± 0.68
Hematocrit (%)	40.22 ± 0.55	33.20 ± 1.70	34.07 ± 1.93	41.55 ± 0.52^*∗*^	47.80 ± 1.50^*∗∗∗*^	38.47 ± 0.58	39.20 ± 1.55
MCV (fL)	67.24 ± 1.36	72.00 ± 1.84#	71.87 ± 0.48	67.70 ± 1.17	70.70 ± 1.78	73.40 ± 0.86	69.65 ± 1.91
MPV (fL)	7.04 ± 0.66	8.60 ± 1.41#	9.43 ± 0.57	6.17 ± 0.74^*∗∗∗*^	6.40 ± 0.36^*∗∗∗*^	8.63 ± 0.05	8.70 ± 0.71
Platelets (×10^3^ *µ*L^−1^)	584.20 ± 56.15	590.00 ± 22.63	631.50 ± 65.73	659.00 ± 41.72	614.00 ± 77.48	426.00 ± 36.74	543.00 ± 42.43
Hemoglobin (g/dL)	11.84 ± 1.11	10.95 ± 0.07	10.00 ± 0.08	11.97 ± 0.17	14.30 ± 0.27	10.97 ± 0.05	10.60 ± 0.42
MCHC (g/dL)	29.40 ± 1.78	28.25 ± 0.21	24.97 ± 0.61^*∗*^	28.85 ± 1.50	30.00 ± 2.33	26.73 ± 1.17	27.05 ± 0.64

NOR = normal control (animals received only saline solution 0.9%); DEN = negative control (animals received N-diethylnitrosamine + saline 0.9%); DOX + DEN = positive control (animals received doxorubicin + N-diethylnitrosamine); GRP I + DEN = animals received polysaccharide fraction GRP I + N-diethylnitrosamine at doses 125 and 250 mg/kg; GRP II + DEN = animals received polysaccharide fraction GRP II + N-diethylnitrosamine at doses 125 and 250 mg/kg. Values represent means ± SD (n = 9). All experimental animals except the normal group (NOR) were exposed to a daily dose of N-diethylnitrosamine (100 mg/kg) for 10 weeks. ^###^*P* < 0.001 and ^##^*P* < 0.01 compared to the normal group; ^#^*P* < 0.05 compared to the normal group; ^*∗*^*P* < 0.05 compared to the negative control; ^*∗∗*^*P* < 0.01 compared to the negative control; ^*∗∗∗*^*P* < 0.001 compared to the negative control.

## Data Availability

The data used to support the findings of this study are included within the article.
